# Insight into over Repair of Hot Carrier Degradation by GIDL Current in Si p-FinFETs Using Ultra-Fast Measurement Technique

**DOI:** 10.3390/nano13071259

**Published:** 2023-04-03

**Authors:** Hao Chang, Guilei Wang, Hong Yang, Qianqian Liu, Longda Zhou, Zhigang Ji, Ruixi Yu, Zhenhua Wu, Huaxiang Yin, Anyan Du, Junfeng Li, Jun Luo, Chao Zhao, Wenwu Wang

**Affiliations:** 1 Key Laboratory of Microelectronics Devices & Integrated Technology, Institute of Microelectronics, Chinese Academy of Sciences, Beijing 100029, China; changhao@ime.ac.cn (H.C.);; 2 Microelectronics Institute, University of Chinese Academy of Sciences, Beijing 100049, China; 3 Process Integration, Beijing Superstring Academy of Memory Technology, Beijing 100176, China; 4 National Key Laboratory of Science and Technology on Micro/Nano Fabrication, Shanghai Jiaotong University, Shanghai 200240, China

**Keywords:** reliability, hot carrier degradation (HCD), Si p-FinFETs, gate-induced drain leakage (GIDL), recovery, oxide trap generation, energy distribution

## Abstract

In this article, an experimental study on the gate-induced drain leakage (GIDL) current repairing worst hot carrier degradation (HCD) in Si p-FinFETs is investigated with the aid of an ultra-fast measurement (UFM) technique (~30 μs). It is found that increasing GIDL bias from 3 V to 4 V achieves a 114.7% V_T_ recovery ratio from HCD. This over-repair phenomenon of HCD by UFM GIDL is deeply discussed through oxide trap behaviors. When the applied gate-to-drain GIDL bias reaches 4 V, a significant electron trapping and interface trap generation of the fresh device with GIDL repair is observed, which greatly contributes to the approximate 114.7% over-repair V_T_ ratio of the device under worst HCD stress (−2.0 V, 200 s). Based on the TCAD simulation results, the increase in the vertical electric field on the surface of the channel oxide layer is the direct cause of an extraordinary electron trapping effect accompanied by the over-repair phenomenon. Under a high positive electric field, a part of channel electrons is captured by oxide traps in the gate dielectric, leading to further V_T_ recovery. Through the discharge-based multi-pulse (DMP) technique, the energy distribution of oxide traps after GIDL recovery is obtained. It is found that over-repair results in a 34% increment in oxide traps around the conduction energy band (E*_c_*) of silicon, which corresponds to a higher stabilized V_T_ shift under multi-cycle HCD-GIDL tests. The results provide a trap-based understanding of the transistor repairing technique, which could provide guidance for the reliable long-term operation of ICs.

## 1. Introduction

DRAM (dynamic random access memory) is one of the core components of electronic equipment, and it has become increasingly important in terms of the development of the information society [1]. For higher memory density, the downscaling of transistor size has become an inevitable trend. Fin Field-Effect Transistors (FinFETs) are recognized as one of the most promising structures for Future DRAM Peripheral Circuits and have been proposed for low-power and high-performance applications beyond 22-nm technology nodes [2,3]. 

With continuous channel length scaling, hot carrier degradation (HCD) has emerged as a major reliability issue in FinFETs [4]. From the trap-based research in HCD, both interface and oxide traps contribute to the overall degradation [5]. A modified compact model and trap spatial distribution investigations facilitate the accurate characterization of HCD [6,7,8]. To characterize the trap generation during HCD stress, a discharge-based multi-pulse technique (DMP) was introduced, which is accessible to oxide traps within and beyond the bandgap [9,10].

To ensure the reliable long-term operation of the transistor in ICs, controlling gate oxide quality is one of the most critical challenges. Several methods have been developed to recover the hot-carrier-induced damage. From the wafer-level view, the forming gas annealing (FGA) process utilizes high-pressure hydrogen or deuterium to passivate the dangling bonds at the interface and thus suppress the interface trap generation (ΔN_IT_) [11,12]. The recovery effects exhibit a positive correlation with annealing temperature, which could be attributed to the thermally activated interface traps discharging [13,14]. From the transistor-level view, an electrothermal annealing (ETA) method shows its feasibility in curing degraded gate oxide. The early ETA method achieves thermal annealing by external micro-heaters but may cause over-heating in metal interconnections due to heat diffusion [15]. Another implementation utilizes the Joule heat inherently generated in the device by the flowing current as the heat source, such as Punch-through and GIDL currents [16,17]. The new methods show superior annealing selectivity and can cure the target transistor, which has experienced severe HCD [18]. This active recovery capability enables ETA to demonstrate better applicability in actual circuits [19]. However, previous ETA methods mainly focus on SOI and GAAFET structures, which themselves have poor heat dissipation performance. In a recent study, the GIDL repairing method showed its feasibility in Bulk FinFETs, which is motivated by field-assisted discharging [20].

However, the trap behaviors during the GIDL repairing process for HCD still remain to be discussed. Another issue that needs to be discussed is the underlying mechanism of the observed V_T_ over-recovery phenomenon. With the development of the ultra-fast measurement technique (UFM), the recovery effect during I_D_-V_G_ measurement is effectively suppressed, further improving the accuracy in HCD characterization [21,22]. In this article, the UFM with a microsecond (~30 μs) delay is used for device characterization. The recovery behaviors of p-FinFETs with 100 nm gate length are investigated. Additionally, the trap behaviors during GIDL repair are discussed with the aid of DMP [23,24]. With the aid of technology computer-aided design (TCAD) tools, the mechanism of over-repairing is explained from the perspective of electric-field simulation. The results provide experimental evidence of the GIDL recovery-related traps and their energy locations, which could provide further understanding of FET recovery techniques.

The remainder of this manuscript is organized as follows. Section 2 elucidates the device under test, the measurement methods, and the TCAD simulation setup. The test results with discussions are shown in Section 3. The conclusions are summarized in Section 4.

## 2. Materials and Methods

### 2.1. Device Fabrication

**Figure 1:**
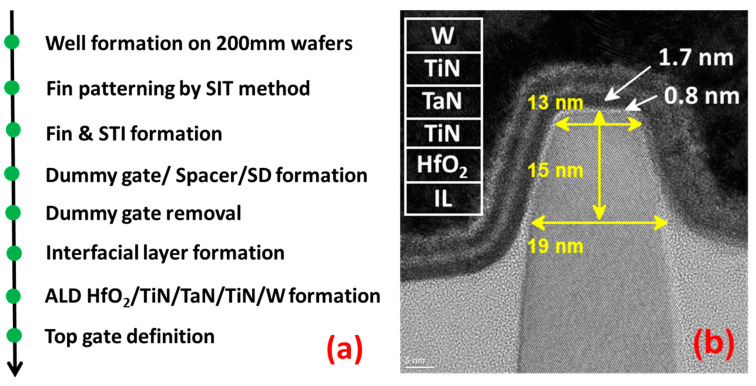
(**a**) The process of p-FinFET with replacement metal gate (RMG). (**b**) TEM image of p-FinFET across channel direction.

The replacement metal gate (RMG) Si bulk p-FinFETs are fabricated using a fully-gate-last process. The equivalent oxide thickness (EOT) is 0.92 nm. The major steps for gate stack formation are shown in Figure 1a: (1) dummy poly-Si/SiO_2_ gate removal; (2) the growth of the 0.8 nm interface layer (IL) of SiO_2_ through chemical oxidation of O_3_; (3) the atom layer deposition (ALD) of 1.7 nm HfO_2_ as a high-k layer; (4) 450 °C post-deposition annealing (PDA); and (5) the deposition of a multi-layer gate stack including ALD Titanium Nitride (TiN)/ ALD Tantalum Nitride (TaN) /CVD Titanium Nitride (TiN)/ ALD Tungsten (W). Figure 1b is the transmission electron microscope (TEM) image of FinFET across the channel direction. P-FinFETs with 100 nm gate length are used for electrical measurements.

### 2.2. Electrical Measurements

Devices are stressed under the worst HCD condition (V_G,STR_ = V_D,STR_) [6]; then, a GIDL voltage is applied to the stressed devices using a synchronized pulse of specified gate (V_G,GIDL_) and drain GIDL biases (V_D,GIDL_). In this article, the Keysight B1530 semiconductor analyzer is utilized to perform UFM of I_D_-V_G_ characteristics using pulse-IV measurements with a duration of 30 μs [22]. Time evolutions of threshold voltage shift (ΔV_T_) are obtained in measure-stress-measure (MSM) mode [25]. V_T_ is extracted through the constant current method with the target linear drain current (I_D,LIN_) of 100 nA × W/L [26]; here, W and L are the gate width and length, respectively.

**Figure 2:**
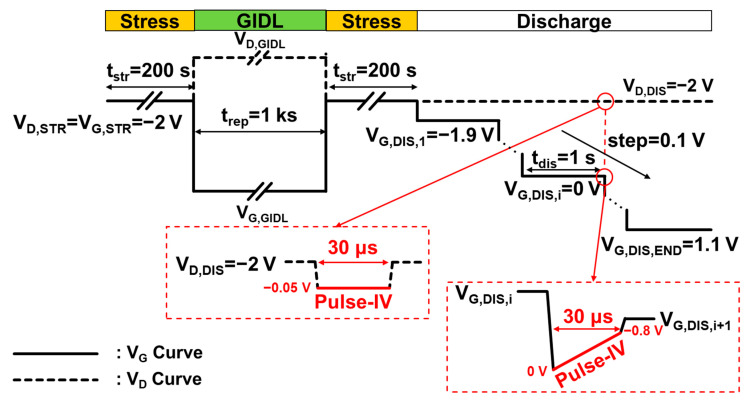
V_D_ and V_G_ test waveforms for the DMP test. Solid curve: V_G_ test waveform. Dashed curve: V_D_ test waveform. Red curve: Pulse-IV measurement.

The DMP experiments are performed to investigate the energy distribution of generated oxide traps during GIDL repair. Figure 2 shows the DMP test procedure used in this work. After 200 s HCD stress, 1 ks GIDL bias is applied to repair the aged device. Afterward, the repaired device is applied with the same HCD stress again; then, V_G_ is sequentially decreased to multiple gate discharge voltage (V_G,DIS_) levels, while V_D_ remains at the stress bias. Each discharge period lasts for only 1 s; then, a pulse-IV is performed to extract ΔV_T_. The overdrive voltage (V_OV_) is calculated by V_OV_ = V_G,DIS_—V_T_; then, the ΔV_T_ ~ V_OV_ relationship is obtained to extract the energy distribution of oxide traps. All of the test results are averaged by a group of three devices at 125 °C.

### 2.3. TCAD Simulation Setup

**Figure 3:**
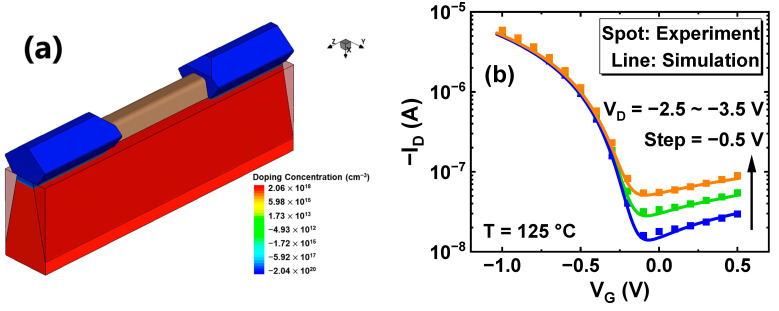
(**a**) 3-D simulation structures of Bulk FinFET with 100 nm gate length. (**b**) TCAD calibration of Bulk FinFET I_D_-V_G_ and GIDL characteristics with experimental data. Orange curve: V_D_ = −3.5 V. Green curve: V_D_ = −3.0 V. Blue curve: V_D_ = −2.5 V.

To bring a further physical explanation, Sentaurus TCAD tools are employed to solve the electric field distribution of p-FinFETs under GIDL repair. The 3-D simulation structure of p-FinFETs with the same Fin shape in TEM is shown in Figure 3a. As shown in Figure 3b, the simulated I_D_-V_G_ curve with GIDL is in good agreement with measured data within 1 m V_T_. The key simulation parameters, such as work function, stress, and the S/D distribution resistance, are concluded in Table 1. Here, the Nonlocal-Path model is used as a band-to-band physical model to accurately match GIDL characteristics in TCAD simulation [27].

## 3. Results and Discussion

### 3.1. Repairing HCD by UFM GIDL

**Figure 4:**
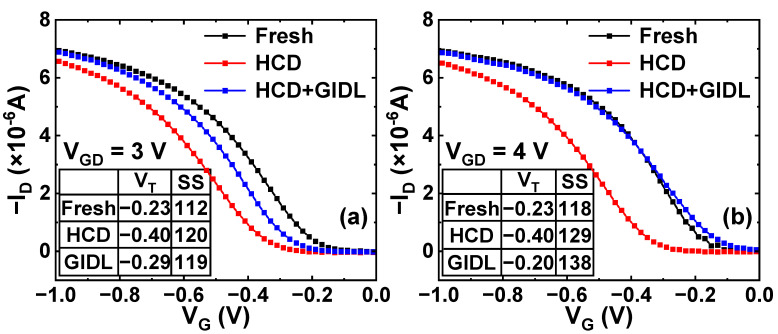
Measured pulse-IV characteristics of p-FinFETs at the initial state, after 200 s HCD, and after 1 ks GIDL repair for (**a**) V_GD_ = 3 V: V_G,GIDL_ = 0.5 V, V_D,GIDL_ = −2.5 V and (**b**) V_GD_ = 4 V: V_G,GIDL_ = 1.5 V, V_D,GIDL_ = −2.5 V. HCD stress: V_G,STR_ = V_D,STR_ = −2 V.

Figure 4 shows that p-FinFETs with 100 nm gate length are subjected to a −2 V HCD stress for 200 s, resulting in a V_T_ degradation of approximately 170 mV and a shift of 8 mV/dec in subthreshold swing (SS). Subsequently, the UFM GIDL repairing process is implemented for 1 ks with different repairing biases. Under moderate GIDL bias of V_GD_ = 3 V (V_G,GIDL_ = 0.5 V, V_D,GIDL_ = −2.5 V), the V_T_ shift is reduced to 60 mV, and the SS shift is negligible, corresponding to a 62.7% recovery ratio, as shown in Figure 4a. In Figure 4b, under a high GIDL bias of V_GD_ = 4 V (V_G,GIDL_ = 1.5 V, V_D,GIDL_ = −2.5 V), the V_T_ recovery ratio is increased to 114.7%, which indicates that the degraded device is over-repaired by GIDL repair. However, this improvement comes at the cost of degradation in SS of 9 mV/dec. Considering that SS degradation reflects the generation rate of interface traps [28], excessive gate-to-drain electric field may result in extraordinary interface damage to the device [29].

### 3.2. Physical Explainations of GIDL Repairing Mechanism

**Figure 5:**
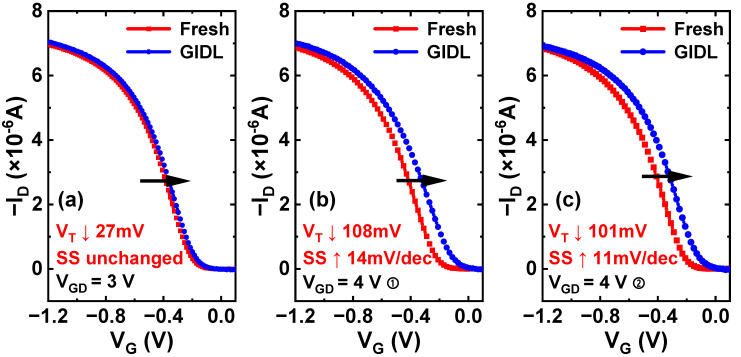
Pulse-IV curves of fresh p-FinFETs before and after 1 ks GIDL biases for (**a**) V_GD_ = 3 V: V_G,GIDL_ = 0.5 V, V_D,GIDL_ = −2.5 V; (**b**) V_GD_ = 4 V①: V_G,GIDL_ = 1.5 V, V_D,GIDL_ = −2.5 V; and (**c**) V_GD_ = 4 V②: V_G,GIDL_ = 0.5 V, V_D,GIDL_ = −3.5 V.

To explain the over-repair phenomenon that occurs at high GIDL biases, a group of fresh devices is subjected to 1 ks GIDL biases, as shown in Figure 5. When a moderate GIDL bias (V_G,GIDL_ = 0.5 V, V_D,GIDL_ = −2.5 V) is applied, the recovery of V_T_ is relatively low, measuring approximately 27 mV. However, under high GIDL biases (by either increasing V_G,GIDL_ or V_D,GIDL_), the recovery of V_T_ exceeds 100 mV. It can be seen that at high gate-to-drain electric fields, a large number of electrons are injected into the gate oxide, which greatly contributes to V_T_ recovery [30]. Moreover, after applying a high GIDL bias, a degradation of approximately 12 mV/dec in SS can be observed. However, no SS degradation is observed at a moderate GIDL bias. 

**Figure 6:**
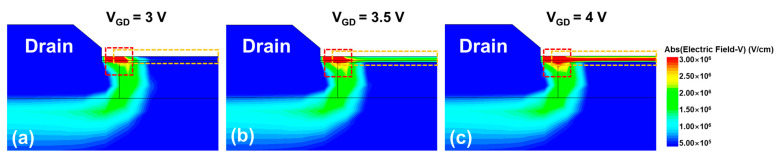
The 2-D view of electric field distributions under GIDL repairing process of (**a**) V_GD_ = 3.0 V, (**b**) V_GD_ = 3.5 V, and (**c**) V_GD_ = 4.0 V.

**Figure 7:**
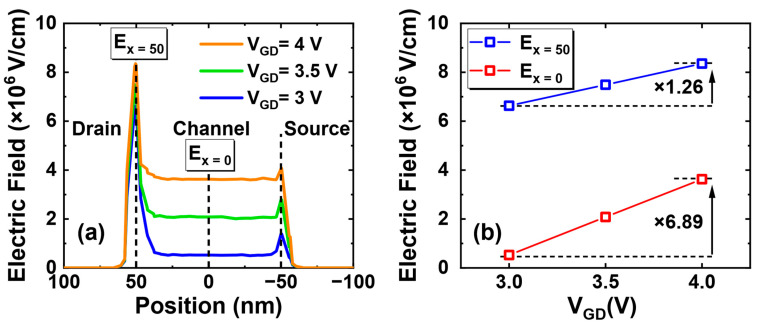
(**a**) Electric field distribution along the surface of the oxide layer in 2-D view under different GIDL biases (V_GD_ = 3.0 V/3.5 V/4.0 V). (**b**) Extracted E_x = 50_ and E_x = 0_ values at different GIDL biases.

Furthermore, a physical explanation is given with the aid of TCAD simulation. The electric field distributions of the device under different GIDL biases are simulated. Cutting vertically the center of gate along the channel direction, the 2-D view of the electric field distributions is shown in Figure 6. As V_GD_ increases from 3 V to 4 V, the channel electric field changes significantly at two positions: one near the drain region, and the other along the channel surface. The 1-D electric field, cut along the surface of oxide layer in 2-D view in Figure 6, is shown in Figure 7a. It is shown that the electric field peaks near the drain region. Then, this peak electric field and the channel center electric field are extracted in Figure 7b and named as E_x = 50_ and E_x = 0_ according to their positional coordinates. 

**Figure 8:**
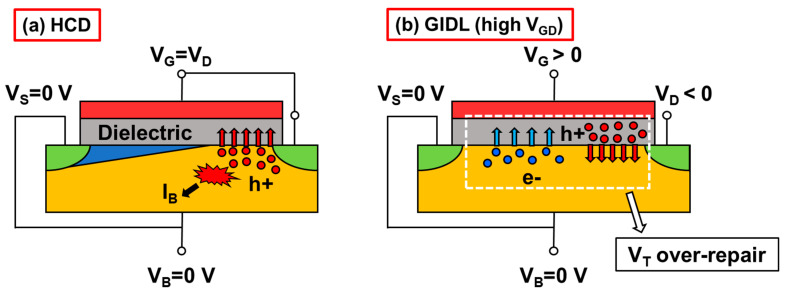
Schematic diagrams during (**a**) HCD and (**b**) GIDL repair with high V_GD_. H+ and e- are short for holes and electrons in the channel, respectively.

As V_GD_ increases from 3 V to 4 V, E_x = 50_ and E_x = 0_ increase by 1.26 and 6.89 times, respectively. As shown in Figure 8, the influences of these two electric fields will be discussed separately. During HCD stress, the channel hot holes near the drain region are captured by the oxide traps in gate dielectric, resulting in the V_T_ degradation. When a GIDL bias is applied, the strong positive electric field near the drain region promotes the trapped holes to be released, which results in V_T_ recovery [31]. The higher E_x = 50_ at V_GD_ = 4 V corresponds to the higher V_T_ recovery ratio (114.7%) in Figure 4. While a high V_GD_ is also like applying a PBTI stress to the device, part of electrons in the channel are captured by the HfO_2_ traps, which also results in the recovery in V_T_ [32]. The higher E_x = 0_ at V_GD_ = 4 V is the main cause of over 100 mV V_T_ recovery in Figure 5. More discharged holes and trapped electrons directly lead to the over-recovery phenomenon observed at high GIDL biases.

### 3.3. Oxide Trap Behaviors during GIDL Repairing

**Figure 9:**
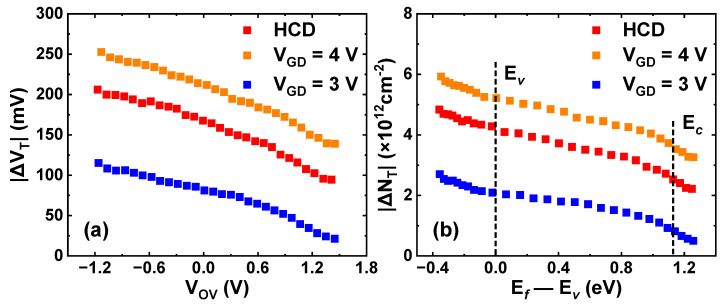
Extracted (**a**) ΔV_T_ ~ V_OV_ and (**b**) ΔN_T_ ~ (E*_f_*—E*_v_*) relationships after applying a new round of HCD stress to recovered devices by different GIDL biases. V_GD_ = 3 V: V_G,GIDL_ = 0.5 V, V_D,GIDL_ = −2.5 V. V_GD_ = 4 V: V_G,GIDL_ = 1.5 V, and V_D,GIDL_ = −2.5 V.

**Figure 10:**
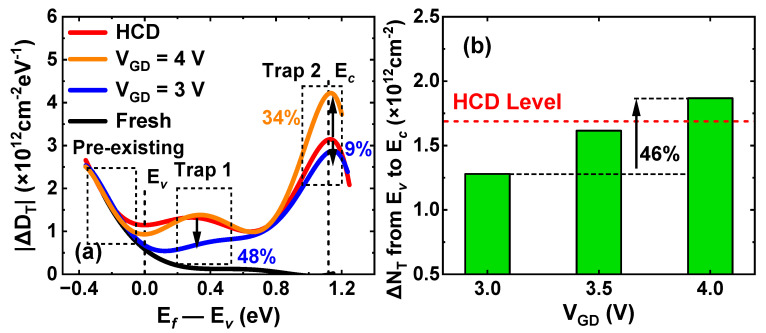
(**a**) Extracted energy distribution of ΔD_T_ in the new round of HCD stress after repairing by different GIDL biases. “Fresh” is ΔD_HT_ extracted by multi-DMP method separately. (**b**) Extracted ΔN_T_ from E*_v_* to E*_c_* in the new round of HCD stress. Fixed V_D,GIDL_ = −2.5 V; V_G,GIDL_ varies from 0.5 V to 1.5 V.

To further analyze the trap behaviors during the GIDL repairing process, DMP experiments are carried out. A group of fresh p-FinFETs is subjected to −2 V HCD stress for 200 s, followed by a 1 ks GIDL repairing process at different GIDL biases. The repaired devices are again applied with the same HCD stress and then discharged following the waveform in Figure 2. Additionally, ΔV_T_ ~ V_OV_ relationships under different GIDL biases are obtained in Figure 9a. Then, ΔV_T_ is converted into the effective trap density (ΔN_T_) following the equation ΔN_T_ = |ΔV_T_| × C_ox_ / q [23]. V_OV_ is converted into the corresponding energy level E*_f_* relative to E*_v_*, i.e., E*_f_*—E*_v_*. Additionally, the ΔN_T_ ~ (E*_f_—*E*_v_*) relationships under different GIDL biases are obtained in Figure 9b. Finally, the energy density of ΔN_T_ (ΔD_T_) is obtained by differentiating ΔN_T_ against E*_f_—*E*_v_*, and the results are shown in Figure 10a. The detailed processing flow can be seen in the previous research [10]. For comparison, a group of devices under the same HCD stress are directly discharged without GIDL repair. Additionally, the energy density of pre-existing traps (ΔD_HT_) is extracted through the multi-DMP method separately, which is represented by a black curve [24].

As can be seen from Figure 10a, the overall degradation of HCD can be divided into three components: pre-existing traps below E*_v_*, and two types of generated oxide traps in the bandgap. Generated trap 1 is at 0.2 eV below the mid-gap of silicon, and trap 2 is located around E*_c_* of silicon. The pre-existing traps are mainly induced by the fabrication process and cannot be repaired by GIDL bias. While generated traps show clear dependence with applied GIDL biases. At a moderate GIDL bias of V_GD_ = 3 V, when a new round of HCD stress is applied, the ΔD_T_ of both trap 1 and trap 2 are reduced by 48% and 9% compared to that under HCD, respectively. While at a high GIDL bias of V_GD_ = 4 V, the ΔD_T_ of trap 2 is 34% higher than that under HCD. In addition, the energy position of trap 1 tends to approach E*_c_* after repair, while the position of trap 2 remains unchanged. To make a clear comparison, ΔD_T_ was integrated between E*_v_* and E*_c_* to extract ΔN_T_, as shown in Figure 10b. As V_GD_ increases from 3 V to 4 V, ΔN_T_ is increased by 46%. It can be seen that, after applying a moderate GIDL bias, the oxide trap density is effectively reduced in the next round of HCD stress. However, at high GIDL biases, although more generated oxide traps are discharged after repair, they are re-charged in the next round of HCD stress, which results in the further generation of oxide trap 2.

**Figure 11:**
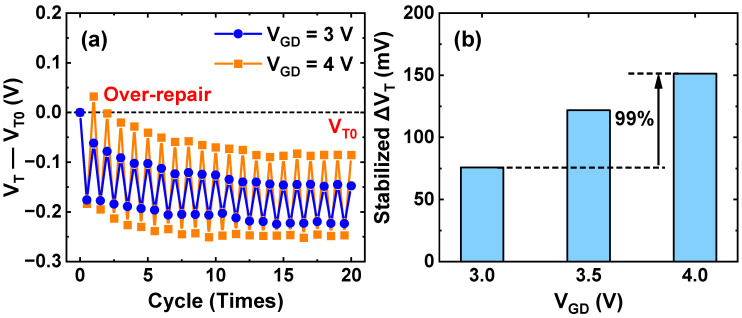
(**a**) Evolutions of V_T_ variation (V_T_—V_T0_) during 20 cycles of HCD/GIDL at different GIDL biases. Each cycle contains 200 s HCD stress (V_G,STR_ = V_D,STR_ = −2 V) followed by 1 ks GIDL repair. (**b**) Stabilized ΔV_T_ during 20 cycles of HCD/GIDL at different GIDL biases. Fixed V_D,GIDL_ = −2.5 V; V_G,GIDL_ varies from 0.5 V to 1.5 V.

To verify the effectiveness of different GIDL biases on long-term HCD recovery, multiple cycles of HCD and GIDL are applied, and the results are shown in Figure 11a. At a high GIDL bias of V_GD_ = 4 V, the repaired device exhibits a lower V_T_ (below the pre-stress value) but shows more severe degradation in the next round of HCD stress. As the HCD/GIDL cycle progressed, the degraded ΔV_T_ and recovered ΔV_T_ in each cycle gradually tended to be the same. As can be seen from Figure 11b, the stabilized ΔV_T_ at high GIDL bias is almost 99.4% higher than that at moderate GIDL bias, which corresponds to the higher ΔN_T_ at high GIDL bias in Figure 10b. The consistent trends between ΔN_T_ and ΔV_T_ illustrate that the cyclic charge–discharge behavior of oxide traps results in the dynamic equilibrium between V_T_ degradation and recovery. To ensure the reliable operation of p-FinFETs under long-term HCD stress, moderate GIDL bias is recommended to ensure the minimum ΔV_T_ under cyclic stress.

**Figure 12:**
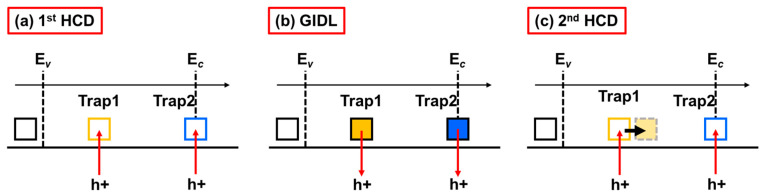
Energy level diagrams during (**a**) the first round of HCD stress, (**b**) GIDL repairing process, and (**c**) the second round of HCD stress.

Finally, the energy level diagrams of the two generated traps are given in Figure 12. As described in the As-grown-generation (AG) model [33], two types of oxide traps with different discharge characteristics have been observed in the silicon energy band. One of them captures a hole without energy level changing, which corresponds to trap 2 around E*_c_*. Due to the shallow energy level of trap 2, it will be charged first during the second round of HCD stress, which corresponds to the higher ΔD_T_ peak observed in Figure 10a. While trap 1 is a type of energy alternating defects (EADs), the energy position shifts towards E*_c_* after capturing a hole. Trap 1 is located at a relatively deep level (0.2 eV below mid-gap of silicon), which will be fully charged only after a complete discharge. Therefore, under a moderate GIDL bias of V_GD_ = 3 V, the ΔD_T_ of trap 1 shows a 48% decrease compared to HCD. For the convenience of reading, all abbreviations and notations frequently used in this article can be seen in Table 2.

## 4. Conclusions

In this article, the GIDL repairing process is carried out with the UFM technique, and trap generations during GIDL repair are experimentally investigated. At a high GIDL bias of V_GD_ = 4 V, the V_T_ recovery ratio reaches 114.7%. With a 6.89 times increase in the channel electric field, more PBTI components are introduced at high V_GD_, which are responsible for additional electron trapping and interface trap generation. At a moderate GIDL bias of V_GD_ = 3 V, the effective density of generated oxide trap 1 (at 0.2 eV below mid-gap of silicon) and trap 2 (around E*_c_* of silicon) are reduced by 48% and 9% in the next round of HCD stress. However, a high GIDL bias will lead to 34% further generation of trap 2. Furthermore, two generated traps show different charge–discharge properties, which corresponds to two types of oxide traps described in AG model. After multiple cycles of HCD/GIDL tests, the degraded and recovered V_T_ reaches the same. The dynamic equilibrium between V_T_ degradation and recovery can be attributed to the cyclic charge–discharge behaviors of oxide traps. To ensure long-term HCD reliability, a moderate GIDL bias is recommended.

## Figures and Tables

**Table 1:** Key device parameters used for TCAD simulation.

Parameter	Bulk FinFET
Channel length (L_G_)	100 nm
Fin height	15 nm
Fin top width	13 nm
Fin bottom width	19 nm
Effective oxide thickness (EOT)	0.92 nm
Source/drain doping	2 × 10^20^/cm^3^
Gate work function	4.97 eV
Source/drain distribution resistance	4.4 × 10^−8^ Ωcm^2^

**Table 2:** List of abbreviations and notations frequently used in this article.

Abbreviations/Notations	Meaning
HCD	Hot carrier degradation
GIDL	Gate-induced drain leakage
FinFETs	Fin field-effect transistors
UFM	Ultra-fast measurement
DMP	Discharge-based multi-pulse technique
SS	Subthreshold swing
PBTI	Positive bias temperature instability
TCAD	Technology computer-aided design
ΔV_T_	Threshold voltage shift
V_G,STR_/ V_D,STR_	Gate/drain stress voltage
V_G,GIDL_/ V_D,GIDL_	Gate/drain GIDL repair bias
V_GD_	Gate-to-drain bias
V_OV_	Overdrive voltage
E_x = 50_	Channel electric field peak near the drain region (at x = 50)
E_x = 0_	Channel center electric field (at x = 0)
ΔN_T_	Effective trap density
ΔD_T_	Energy density of ΔN_T_
E*_v_*/ E*_c_*	Valance/conduction energy band of silicon

## Data Availability

The data may be obtained from the authors upon request.
